# The Interplay between Campylobacter and the Caecal Microbial Community of Commercial Broiler Chickens over Time

**DOI:** 10.3390/microorganisms9020221

**Published:** 2021-01-22

**Authors:** Ilaria Patuzzi, Massimiliano Orsini, Veronica Cibin, Sara Petrin, Eleonora Mastrorilli, Alessia Tiengo, Federica Gobbo, Salvatore Catania, Lisa Barco, Antonia Ricci, Carmen Losasso

**Affiliations:** 1 Microbial Ecology and Genomics Laboratory, Istituto Zooprofilattico Sperimentale delle Venezie, Viale dell’Università 10, 35120 Legnaro, Italy; ilaria.patuzzi@eubiome.it (I.P.); MOrsini@izsvenezie.it (M.O.); spetrin@izsvenezie.it (S.P.); eleonora.mastrorilli@embl.de (E.M.); 2 National Reference Laboratory for Salmonella, Istituto Zooprofilattico Sperimentale delle Venezie, Viale dell’Università 10, 35120 Legnaro, Italy; vcibin@izsvenezie.it (V.C.); atiengo@izsvenezie.it (A.T.); aricci@izsvenezie.it (A.R.); 3 Avian Pathology Laboratory, Istituto Zooprofilattico Sperimentale delle Venezie, Viale dell’Università 10, 35120 Legnaro, Italy; fgobbo@izsvenezie.it (F.G.); scatania@izsvenezie.it (S.C.); 4 Experimental Microbiology Department, Istituto Zooprofilattico Sperimentale delle Venezie, Viale dell’Università 10, 35120 Legnaro, Italy; lbarco@izsvenezie.it

**Keywords:** *Campylobacter jejuni*, poultry microbiota, metataxonomics

## Abstract

*Campylobacter* is the most frequent foodborne zoonotic bacteria worldwide, with chicken meat being overwhelmingly the most important reservoir for human infections. Control measures implemented at the farm level (i.e., biosecurity or vaccination), which have been successfully applied to limit other pathogens, such as *Salmonella*, have not been effective in reducing *Campylobacter* occurrence. Thus, new approaches are needed to fully understand the ecological interactions of *Campylobacter* with host animals to effectively comprehend its epidemiology. The objective of this study was to analyse longitudinally the gut microbiota composition of *Campylobacter*-infected and non-infected farms to identify any difference that could potentially be indicative of gut colonization by *Campylobacter* spp. Differences in the colonization rate and timing were observed at the farms that became positive for *Campylobacter jejuni* over the investigated time points, even though in positive tests, the occurrence of *Campylobacter jejuni* gut colonization was not observed before the second week of the life of the birds. Significant differences were observed in the abundances of specific bacterial taxa between the microbiota of individuals belonging to farms that became *Campylobacter* positive during the study and those who remained negative with particular reference to *Bacteroidales* and *Clostridiales*, respectively. Moreover, *Campylobacter* colonization dramatically influenced the microbiota richness, although to a different extent depending on the infection timing. Finally, a key role of *Faecalibacterium* and *Lactobacillus* genera on the *Campylobacter* microbial network was observed. Understanding the ecology of the *Campylobacter* interaction with host microbiota during infection could support novel approaches for broiler microbial barrier restoration. Therefore, evidence obtained through this study can be used to identify options to reduce the incidence of infection at a primary production level based on the targeted influence of the intestinal microbiota, thus helping develop new control strategies in order to mitigate the risk of human exposure to *Campylobacter* by chicken meat consumption.

## 1. Introduction

Thermotolerant Campylobacters constitute the most frequent bacterial cause of intestinal infection in many developed countries [1]. In 2018, *Campylobacter* was the most commonly reported gastrointestinal bacterial pathogen in humans in the European Union (EU), particularly the species *C*. *jejuni* and *C*. *coli*, with 246,571 confirmed cases of campylobacteriosis, corresponding to an EU notification rate of 64.1 per 100,000 population [2]. Such significant numbers of Campylobacter infections have been recorded since 2005, and numbers are increasing over time [2]. Foods of animal origin, in particular chicken meat, have been identified as significant sources of this enteropathogen [3]

Birds become infected with *Campylobacter* via the faecal-oral route, after which the microorganism colonizes the intestinal tract and establishes itself in the caecum, the organ that carries the highest loads of this pathogen [4]. Campylobacter also colonizes the small intestine and, to a lesser extent, liver, spleen, deep muscle, thymus, and bursa of Fabricius [5,6,7]. *C. jejuni* and *C. coli* are extremely prevalent in poultry production. In the EU, up to 80% of flocks harbour these bacteria, although the exact prevalence varies according to country [2]. Similarly high levels of transference to poultry meat following processing were recognized [8,9]. Different *Campylobacter* subtypes are usually isolated within the same flock as a possible consequence of continuous flows of new *Campylobacter* from different sources entering the poultry flocks. Infection is seldom detected in chickens that are less than 1–2 weeks old, but once *Campylobacter* colonizes an individual animal, transmission occurs rapidly, resulting in infection of nearly the entire flock within a few days [8].

To date, most strategies to reduce *Campylobacter* colonization in chickens and, thus, its release into the farm environment have not been effective [2], to the detriment of food safety. Nevertheless, methods based on the mechanism of competitive exclusion have been widely explored, and several studies demonstrate that modification of intestinal microbiota may be effective in outcompeting *Campylobacter* challenges [10,11]. For the development of effective intervention strategies in this direction, it is important to adequately characterize the intestinal microbiota of chickens [12,13,14,15]. Most of the studies that have been published to date have been based on culture methods, or on relatively simplified molecular biology-based approaches [16]. Conversely, the use of a metagenomic approach has demonstrated efficacy in detailing chicken gut microbiota and relationships between bacteria [12,17].

The main objective of the present work was to explore the dynamic changes of chicken gut microbiota composition occurring during *Campylobacter* infection, with particular emphasis on the first stage of colonization. Although chickens are more likely to harbour *Campylobacter* with age because of rapid changes within their microbial communities, some animals remain negative throughout their entire production cycle. This evidence in turn suggests that some form of microbial community could be associated with protection of the gut from *Campylobacter* colonization. This protective community could be partly maintained in farms that remain *Campylobacter*-free during the whole production cycle. The present work aims to test this hypothesis to support the development of effective control strategies for *Campylobacter* in broilers at the pre-harvest stage.

## 2. Materials and Methods

### 2.1. Sampling Design

*Campylobacter* infection dynamics were investigated in four broiler farms located in Northern Italy (farms 1, 2, 3, and 4) sampled at the same time of the year. These farms utilized the same biosecurity measures, the same diet, antimicrobial(s) usage, and vaccination protocols and were comparable in size and management approaches and housed birds that derived from the same parental lineage (Ross, Aviagen). 

For each farm, one flock among those housed in the same barn during the entire study period was randomly chosen and longitudinally monitored for the entire production cycle. For each farm and sampling event, a total of 16 birds were randomly sampled, for a total of 80 birds for each sampled farm.

Sampling was performed at five different time points (days 7, 14, 21, 28, and 35 after chickens hatched). The sampling scheme was performed following Evans et al. [18]. Birds were anesthetised with Zoletil (0.15 mL/Kg intramuscular administration, Virbac S.r.l. Milano, Italy) and then humanely euthanized with Tanax^®^ (0.3 mL/kg, intravenous administration, MSD Animal Health S.r.l. Milano, Italy), and caecal samples were collected for analysis. From each caecal sample, two independent biological replicates were collected by means of swabs (FecalSwabTM, Copan Diagnostics Inc, Brescia, Italy). Aseptic techniques and disposable equipment were used for each sample. The entire work flow was approved by the IZSVe ethics committee (OpBA project n. 05/2015).

### 2.2. Campylobacter Identification from Caecal Swabs

*Campylobacter* from individual caecal swabs was isolated and identified as follows. Each caecal swab was vortexed with 2 mL swab medium (modified Cary Blair medium, Copan Diagnostics Inc., Brescia, Italy), and a 1 mL volume of caecal suspension was added to 9 mL of Preston Broth (ISO 10272) in a 15 mL test tube. The tube was inverted three times, pierced in the upper part, and incubated under microaerophilic conditions for 48 h at 41.5 ± 1 °C.

After incubation, Preston cultures were checked for the presence of *C. jejuni*, *C. coli,* and *C. lari* by using the Real Time PCR iQ-Check^®^ Campylobacter (Bio-Rad Laboratories, Inc., Hercules, CA, USA) kit according to the producer’s instructions.

Preston Broth cultures that were positive by Real Time PCR were then processed to isolate *Campylobacter*. Briefly, an aliquot of each PCR-positive Preston Broth culture, obtained after incubation of the caecal swab content, was incubated under microaerophilic conditions for an additional 48 h at 41.5 ± 1 °C on Campylobacter Charcoal Differential Agar (CCDA) Petri plates. One typical *Campylobacter* suspected colony was isolated from each plate, transferred to Blood Agar (AS) plates, and incubated under microaerophilic conditions for 48 h at 41.5 ± 1 °C. Positive colonies were typed by PCR according to Denis et al. [19]. *C. jejuni* ATCC 29,428 and *C. coli* 590/2 (belonging to the standard collection of the EU Community Reference Laboratory for *Campylobacter*-CRL Bilthoven, The Netherland) were used as positive controls.

### 2.3. Metataxonomics

For each selected farm, on days when the sampling showed all caeca tested negative or positive for Campylobacter, five samples randomly selected were subjected to metataxonomic investigation. For each sampling day when both positive and negative birds coexisted (day 28: farm 3 and day 14: farm 4), to better intercept changes in the microbiota composition, five extra samples were subjected to metataxonomic investigation. The entire sampling schemes finally accounted for 110 caecal samples (with suitable biological replicates for samples) that were processed. 

#### 2.3.1. DNA Extraction

Total DNA for metataxonomic analysis was extracted using a column-based kit (QIAamp DNA Mini Kit, QIAGEN, Hilden, Germany) starting from 200 µL of caecal content, following the manufacturer’s instruction. Thermal lysis was carried out for 2 h, and RNaseA (100 mg/mL) was added to each sample to ensure RNA-free preparation. Total DNA was resuspended in 200 µL of nuclease-free water and stored at −20 °C until library preparation for sequencing.

#### 2.3.2. 16SrDNA Sequencing

Extracted DNA was used as a template in amplicon PCR to target the hypervariable V3 and V4 regions of the bacterial 16S rRNA gene. The 16S metagenomics library was prepared according to the Illumina 16S Metagenomic sequencing Library Preparation protocol, using the primers Bact341F and Bact785R (Fwd: CCTACGGGNGGCWGCAG and Rev: GACTACHVGGGTATCTAATCC) previously described by Klindworth A et al. [20]. PCR clean-up was performed with Agencourt AMPure XP beads (Beckman Coulter Genomics, Indianapolis, IN, USA). Samples were equimolarly pooled, and sequencing was performed with an Illumina HiSeq2500 sequencer in RAPID (2 × 250 bp) mode. Read sequences were deposited in the Sequence Read Archive (SRA) of the NCBI (http://www.ncbi.nlm.nih.gov/sra) BioProject PRJNA688683.

#### 2.3.3. Read Preprocessing and OTU Table Construction

After sequencing, data underwent a quality control procedure using the FastQC tool [21]. Data were then cleaned by removing adapters, primers, and performing dereplication of sequences using an in-house bash script. In addition, data were filtered based on the quality and length of the reads, so that only data with a quality higher than a given threshold (QPhred ≥ 20) and reads longer than 100 bp were retained. All subsequent steps were performed using python scripts that are part of the QIIME1 pipeline (version 1.9.0) [22]. Data obtained from the filtering step underwent read pairing in order to obtain a single file in which the reads obtained by sequencing the 16S fragments on the forward and reverse strands were joined by their overlapping region. Then, an OTU picking step was performed, assigning reads to a particular taxonomy by directly mapping the same reads to a 16S sequences database (GreenGenes database [23], last release May 2013). Following the technique described in Barruzzo et al. [24], the obtained count table was pre-processed with GMPR tool [25] for normalization and with scImpute [26] for zero-imputation. This was done to minimize sequencing process biases, and a weighted mean over replicates that accounted for sequencing depth was retained for further analysis.

#### 2.3.4. Microbiota Analysis

All the statistical analyses and the visualizations were produced within the *R* environment [27] using appropriate packages (stats [27], ggplot2 [28], DiversitySeq [29], phyloseq [30]). Alpha diversity analysis was performed on the pre-processed count table by means of the DiversitySeq [29] package, choosing the *Observed Richness* index for richness calculation, and the *Pielou* index for evenness. On the same data, we performed Non-Metric Multidimensional Scaling (NMDS) dimensionality reduction using phyloseq [30] functions, choosing the Bray–Curtis distance as a measure for beta diversity. Finally, with the aim of understanding the *Campylobacter* interaction network, we applied the MetaMIS tool [31] to proportional abundances related to farm 4 data, i.e., the farm that first became positive and, consequently, that had sufficiently extended time-series data including both negative and positive samples.

### 2.4. Statistical Analysis

One-way ANOVA was applied to determine differences in caecal microbial communities among the four farms and between *Campylobacter* negative and positive samples when data were normally distributed, as suggested by Zou and co-authors [32]; otherwise a Kruskal–Wallis test was performed; *p*-values < 0.01 were considered statistically significant. In addition, a Kruskal–Wallis test was performed on samples from farms 1 and 2 to test for statistically significant differences in alpha diversity values; *p*-values < 0.05 were considered statistically significant. PERMANOVA tests were conducted (*adonis2* function of the *vegan* R package, version 2.5–7) on the Bray–Curtis distance matrix to test for differences between the two negative farms, between the two positive farms, and between samples related to early and late phases of farm 4 birds’ lives.

## 3. Results

### 3.1. Dynamics of Campylobacter Infection over Time

Time-series analyses were conducted to investigate the dynamics of *Campylobacter* infection at four broiler farms, monitoring them weekly for 35 days after hatching, corresponding to the whole production cycle. Two out of four farms showed *Campylobacter* infection at different time points (farms 3 and 4), while two farms remained negative during the entire time span (farms 1 and 2, Table 1). *C. jejuni* was the only species isolated during the entire study; we did not detect *C. coli* in any of the broiler caeca examined.

### 3.2. Effect of C. jejuni Colonization on the Caecal Microbial Community Composition

Time series metataxonomic analyses were conducted to investigate the changes in birds’ caecal microbiota in relation to the dynamics of *Campylobacter* infection.

A total of 35,393,214 quality controlled sequence reads, with an average length of 443.67 ± 3.29, were resolved into 5410 OTUs. Considering all samples together, the average number of assigned counts was 160,878.245, with a range of 52,762 to 945,882. After cleaning the dataset from archaea and cyanobacteria reads and singletons (i.e., OTUs present in a single caecal sample with count 1), 4010 OTUs distributed among 17 *phyla* remained.

**Figure 1:**
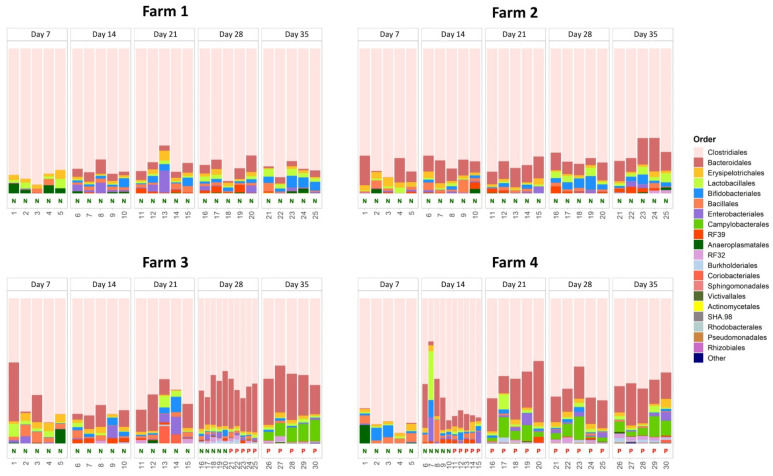
Chicken caeca microbial community composition at the order level. The 20 most proportionally abundant (in mean) orders are explicitly shown, while the remaining portion of microbial contributions is grouped in the “Other” category. Negative samples are labelled with a green N on the *x*-axis, the positive ones with a red P.

The relative abundance of the genus *Campylobacter* in the studied caecal microbiota is reported in the Supplementary Table S1. We observed strict concordance between *Campylobacter* infection in caeca, as assessed by the standard bacterial culture method we used, and the presence of *Campylobacter* DNA in caecal matter, as confirmed by metataxonomics. Figure 1 shows the results obtained for the taxonomic assignment at the order level, for the four studied farms. This representation was chosen as an acceptable compromise between the clarity of graphical representation and the detail of taxonomic information.

After annotation with the GreenGenes database, the most abundant bacterial order at all the selected farms was *Clostridiales*, ranging from 29.8% to 94.0% (median: 76.6%). Caeca from *Campylobacter*-negative and -positive farms showed different microbial community compositions. The relative abundance of *Clostridiales* was higher in caeca from the *Campylobacter*-negative farms than in those from the positive ones (80.0% vs. 65.7%; *p* < 0.01, Kruskal–Wallis test). An opposite result was observed for *Bacteroidales*, an order that was more abundant in caeca from *Campylobacter*-positive farms than in those from the negative ones (17.7% vs. 7.7%; *p* < 0.01, Kruskal–Wallis test). As expected, the order *Campylobacterales* was more abundant in the caeca from the *Campylobacter*-positive farms than in those from the negative ones (2.7% vs. 0.0%; *p* < 0.01, Kruskal–Wallis test). No additional statistically significant differences were observed.

Interestingly, *Clostridiales* and *Bacteroidales* were also different in terms of abundance between the two negative farms. In particular, *Clostridiales* were more abundant in caeca from farm 1 than in those from farm 2 (82.9% vs. 77.2%; *p* < 0.01, one-way ANOVA), while *Bacteroidales* were more abundant in caeca from farm 2 than in those from farm 1 (11.3% vs. 4.1%; *p* < 0.01, one-way ANOVA).

When considering the two positive farms together and comparing negative and positive caeca, some differences emerged. In *Campylobacter*-positive caeca, a lower abundance of *Bacillales* (1.0% vs. 3.2%; *p* < 0.01, Kruskal–Wallis test) and a higher abundance of *Bacteroidales* (21.9% vs. 13.5%; *p* < 0.01, Kruskal–Wallis test) were observed; a similar result was observed for the *Campylobacterales* (5.4% vs. 0%; *p* < 0.01, Kruskal–Wallis test), as expected. No additional remarkable differences were observed. Although not statistically significant, the positive caeca from positive farms harboured several orders at very low abundance, e.g., the *Rhodobacterales*, the *Pseudomonadales*, or the *Rhizobiales* (see Supplementary Table S2).

Generally, three main caecal community composition structures can be ascertained in the studied caecal samples. The first one was typical of birds belonging to farms that remained *Campylobacter*-free during their entire production cycles (farms 1 and 2). In these birds, the *Clostridiales* dominated over other bacterial orders, accounting for more than 75% of the entire microbiota and together with the *Bacteroidales* forming up to 90% of the community. In terms of relative abundance, these two orders were generally followed by a few taxa (*Bifidobacteriales*, *Erysipelotrichales*, *Lactobacillales,* and *Bacillales*), each of them accounting for about 2% of the entire microbial community within the birds’ caeca.

A second scenario was observed in the birds that remained *Campylobacter*-free but belonging to farms that become infected by *C. jejuni* over time (farms 3 and 4). In these birds’ caeca, the most abundant order (the *Clostridiales*) comprised less than the 70% of the entire microbial community, the *Bacteriodales* more than 10%, the *Bifidobacteriales*, *Lactobacillales*, and *Erysipelotrichales* were stable and together accounted for 2% of the entire community, with the *Enterobacteriales* and the *Bacillales* comprising, on average, less than 1% of the entire community.

Finally, a third scenario was identified for *Campylobacter*-positive birds. Here, the microbial communities of caeca from these birds were characterized by *Clostridiales* and *Bacteroidales*, together accounting for more than 80% of the entire community, followed by the *Campylobacterales*, which dominated over the other less abundant orders.

### 3.3. Effect of C. jejuni Colonization on Caecal Microbial Community Diversity

**Figure 2:**
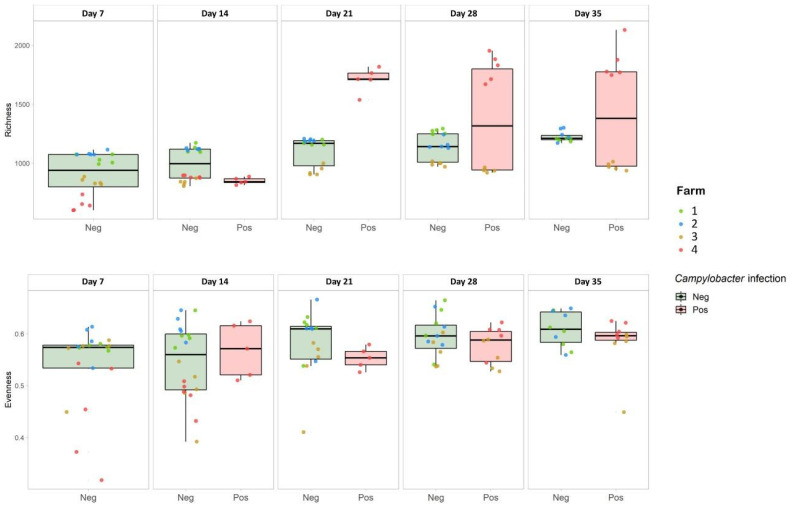
Alpha diversity analysis in terms of OTU richness (**top**) and Pielou index (**bottom**). Results are divided according to whether the sample was positive or negative for *Campylobacter*. Green box plots were constructed from negative samples’ alpha diversity results, red box plots were constructed from positive samples’ alpha diversity results. The single values related to each sample are shown as points colored according to the farm to which they belonged.

Estimates of microbial community diversity were assessed using two measures of alpha-diversity, OTU richness and Pielou index of evenness, and are reported in Figure 2.

As far as OTU richness is concerned, a general increasing trend was observed from day 14 onwards. In addition, caeca from birds belonging to farms 1 and 2, both negative for *Campylobacter* infection over time, displayed steadily increasing OTU richness between day 7 and day 35 of sampling (Kruskal–Wallis test; *p* = 0.0090 and *p* = 0.0088, respectively). For farm 3, a different scenario was observed for the timepoints showing a mixture of *Campylobacter*-positive and –negative samples (days 28 and 35); on these days, the birds that were *Campylobacter*-positive had significantly less diverse microbial communities in their caeca than those in which *Campylobacter* were not detected (*p* < 0.001, one-way ANOVA). For farm 4, instead, the richness observed in the birds’ caeca almost doubled after the occurrence of *Campylobacter* colonization (day 14 onward). Comparing age-matched negative birds belonging both to farms 1 and 2 (negative farms) and farms 3 and 4 (positive farms), the caecal microbial community was significantly more diverse (*p* < 0.001, one-way ANOVA) on days 14, 21, and 28.

Regarding evenness, (as described by the Pielou index) a general steady state was observed for negative farms (1 and 2) while a slight but stable increase over time was noticed for farms 3 and 4, independent of the emergence of *Campylobacter* infection. A general dispersion in the evenness values for the positive farms (3 and 4) was observed on each sampling day.

**Figure 3:**
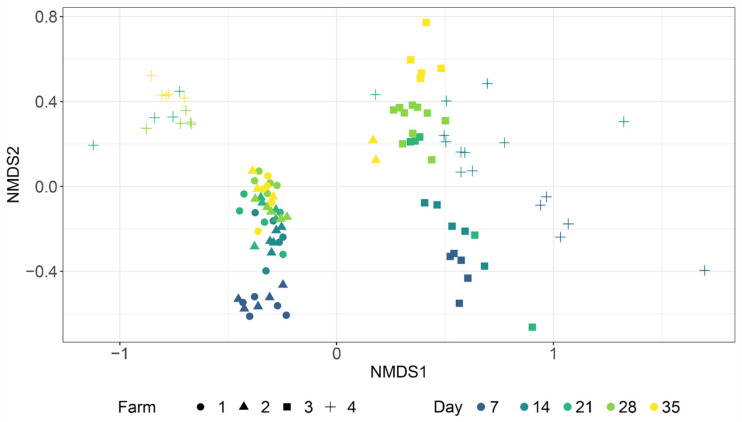
NMDS ordination plot based on the Bray–Curtis distance. Colors reflect the sampling day (from day 7 to day 35), while the shape represents the farm to which the samples belong.

The Non-Metric Multidimensional Scaling (NMSD) plot (Figure 3) between farms and sampling day revealed a close relationship between the microbial caecal communities from non-infected farms (blue and green in the figure), which grouped together in the same cluster and showed a similar and compact variation over time. 

However, microbial caecal communities taken on day 7 deviated from all the others. Moreover, microbial caecal communities from two samples from non-infected farms (collected on day 35) were close to microbial communities of samples collected around the time of infection belonging to *Campylobacter*-positive farms 3 and 4 (day 28 for farm 3 and day 14 for farm 4). The PERMANOVA test run on the Bray–Curtis distance matrix considering both time and farm as factors (interaction model) confirmed that a statistical difference between the two negative farms existed only if including the two above-mentioned outlier samples (*p*-value: 0.0303), and that the significance disappeared when discarding them from the testing procedure (*p*-value: 0.1192). Conversely, the two positive farms showed two completely different behaviors. Microbial caecal communities of birds belonging to farm 3 (yellow samples) showed a temporal trend alongside the NMDS axis 2, that was consistent with the trend observed for negative farms, even though it had a different degree of spatial spread. A different picture was obtained in the case of farm 4, where a dramatic separation resulted in two different clusters within the opposite sides of the NMSD axis 1. The right one contained samples from early sampling (days 7 and 14), before *Campylobacter* infection appeared, while the left one included only samples that were infected with *Campylobacter* (on days 21, 28, and 35) (Figure 3). These observations were confirmed by the PERMANOVA test run on the Bray–Curtis distance matrix considering both time and farm as factors (interaction model), the results of which showed a statistical difference both between the two positive farms (*p*-value: 0.0001) and between samples related to early (days 7 and 14) and late phases (days 21, 28, and 35) of farm 4 birds’ lives (*p*-value: 0.0001).

### 3.4. Effect of C. jejuni Colonization on Caecal Microbial Community Interactions

**Figure 4:**
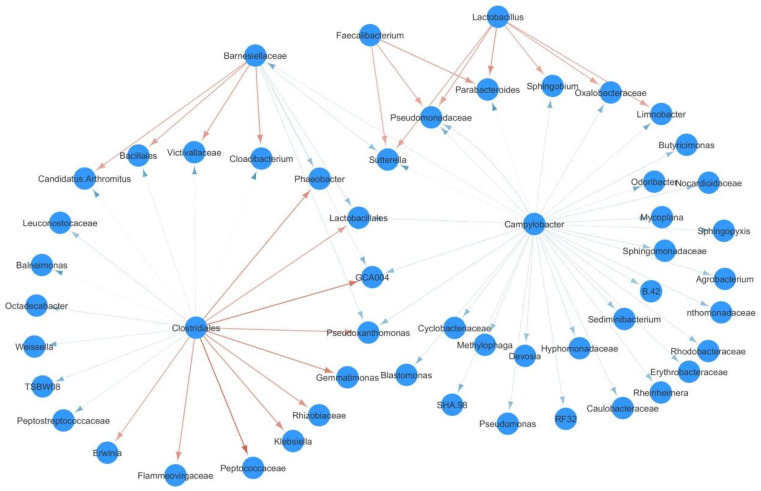
*Campylobacter* interaction network. Red arrows represent positive interactions, while blue arrows represent negative ones.

An ecological network focusing on the top 0.1% OTU interactions was built on the basis of a Lotka–Volterra model for farm 4 with the aim of investigating the relationships between *Campylobacter* and other members of the chickens’ caecal microbial community (Figure 4). 

The network consisted of 53 nodes and 71 edges. The *Campylobacter* genus accounted for 32 negative interactions against an equal number of OTUs classified at different taxonomic levels. Interestingly, *Campylobacter* showed no significant positive interactions. Conversely, several positive interactions were detected from the *Faecalibacterium* and *Lactobacillus* genera towards six other OTUs (*Limnobacter, Parabacteroides, Pseudomonadaceae, Sufferella, Sphingobium* and *Oxalobacteraceae*), which in turn were negatively affected by *Campylobacter*. Similarly, *Clostridiales* positively interacted with *Lactobacillales*, GCA004, and *Pseudoxanthomonas*, which in turn were negatively influenced by *Campylobacter* and *Barneselliaceae*. 

## 4. Discussion

The idea that the microbiota can protect the host from colonization by pathogens, thus leading to colonization resistance, has been the subject of many studies [33], although only a few have generated evidence defining key species involved in the protection of poultry from *Campylobacter* colonization [17,34,35,36,37,38]. In the present work, we described the dynamics of *Campylobacter* colonization over time in the light of its relationships with the caecal microbiota of commercial broilers. Our results reveal several features of *Campylobacter* interaction with the resident microbial community never discussed before, and provide useful insight into how to tackle caecal infection caused by this foodborne pathogen.

First of all, a difference in the colonization rate and timing was observed in the two farms that became positive for *C. jejuni* over the five investigated time points, even though for both farms, the gut colonization by *C. jejuni* was not observed before the second week of the chickens’ life. This phenomenon was already experimentally described [39,40] and is associated with the suppression of the existing *Campylobacter* population by the innate maternal antibodies acquired during the pre-laying period [4,41]. When maternal antibodies are removed from the system, an equilibrium between the pathogen and broiler immune system is reached, and the potential for *Campylobacter* infection is strongly influenced by the pathogen itself and the host’s health, including the microbiota structure. In this regard, the β-diversity results suggest a common microbial signature for gut microbiota belonging to young birds and a change in the microbiota structure that is age related and Campylobacter infection driven. Given the position in the graph of gut microbiota from two birds belonging to Farm 2 (Campylobacter negative), suggesting a community shift towards the typical structure of Campylobacter-positive microbiota (1st Cartesian Quad), we can speculate a possible late *Campylobacter* infection not yet detectable due to the short broiler production cycle. However, we cannot exclude the possibility that the differences in the colonization rate among farms could be related to differences at the *Campylobacter* strain level and/or to the presence of possible co-infections with non-bacterial pathogens (i.e., *Coccidia*) [42]. 

Secondly, we identified significant differences in the abundances of specific bacterial taxa between the individuals belonging to farms that became *Campylobacter*-positive during the study, and those in farms that remained *Campylobacter*-negative, with particular reference to *Bacteroidales* and *Clostridiales*, respectively. However, these differences boiled down to the *Bacteroidales* only, when comparing both negative and positive individuals belonging to *Campylobacter*-positive farms. In this last case, *Bacillales* also differentiated negative and positive birds belonging to those farms. These results suggest that the abundance of specific taxa in the microbiota might reduce the resilience of chicken microbiota towards *Campylobacter* colonization. Similar results were also found in other experimental settings conducted both in humans exposed to *Campylobacter* for occupational reasons [43] and in experimentally infected mice [44,45]. Remarkably, a difference in the abundances of *Clostridiales* and *Bacteroidales* was noticed also between the individuals belonging to the two negative farms, even though the relative abundance of *Clostridiales* was generally greater than that found in the individuals belonging to the positive ones. This suggests some level of inter-individual variability in the abundance of certain taxa among individuals less susceptible to *Campylobacter* colonization.

Thirdly, as far as microbial diversity is concerned, in agreement with previous studies [46,47,48], the richness and the evenness of caecal microbiota were strongly influenced by birds’ age, for both negative and positive farms. In addition, *Campylobacter* colonization dramatically influenced the microbiota richness of positive individuals although to a different extent depending on the timing of the infection. Indeed, a dramatic increase in caecal microbiota richness was noticed only when infection occurred at an early stage in the birds’ life (farm 4, day 14); moreover, the caecal microbiota evolved differently over time, in terms of microbiota diversity, as observed for the two positive farms that showed a different timing of infection. We cannot exclude the possibility of a mutual interplay between *Campylobacter* and the resident microbial community in birds belonging to the two farms, dependent on the *Campylobacter* load.

Lastly, in order to investigate whether the correlation between specific bacterial taxa and susceptibility to *Campylobacter* caecal colonization was due to a direct inhibitory effect of the commensals or to their influence on the caecal milieu, we performed a network analysis to reveal the key role of *Faecalibacterium* and *Lactobacillus* genera in this scenario. Interestingly, these two taxa were not directly contrasted by any other community component but instead, they were found to exert a positive action against six different taxa (i.e., *Limnobacter*, *Parabacteroides*, *Pseudomonadaceae*, *Sutterella*, *Sphingobium,* and *Oxalobacteraceae*) that in turn were exposed to the negative action triggered by *Campylobacter* itself. Thus, these six taxa might be involved in the maintenance of the resilience within the microbial community, under the positive effect of *Faecalibacterium* and *Lactobacillus*. These results are in agreement with previously published studies that describe the competitive reduction of *C. jejuni* obtained by increasing the microbial load of *Lactobacillus*, even though these studies did not investigate specifically the ecological network of *Campylobacter* [49,50,51,52]. A statistically significant association between the increase in *Faecalibacterium* in chicken caeca and *Campylobacter* infection was observed previously [53]. Evidence collected from humans suggests that *Faecalibacterium prausnitzii*, a butyrate-producing bacterium [54], localizes itself near the epithelial cells, as it attaches to the mucous layer [55]. Translating this information to chickens, Thibodeau and colleagues [53] considered the possibility that *Faecalibacterium* shares the same ecological niche with *Campylobacter*. However, the authors failed to determine how *Campylobacter* could positively interact with *Faecalibacterium* and the relative importance of *Faecalibacterium* for chicken intestinal health, as the ability of *Faecalibacterium* to produce butyrate, reported to be detrimental to *C. jejuni* [56], appeared to contradict *Faecalibacterium*’s positive association with *C. jejuni* [53]. Consequently, the current study is the first to reveal and define the mutualistic relationship between these two bacterial genera (*Faecalibacterium* and *Campylobacter*), which occurs by means of an intermediate microbial conglomerate (*Limnobacter*, *Parabacteroides*, *Pseudomonadaceae*, *Sutterella*, *Sphingobium,* and *Oxalobacteraceae*) that appears to modulate their mutual interactions. Moreover, the network topology suggests the possibility of a commensalistic relationship between *Faecalibacterium* and the intermediate microbial conglomerate; conversely, an amensalistic relationship could be in place between the intermediate microbial conglomerate and *Campylobacter*.

Taken together, these findings pave the way for an evidence-based design of customized gut microbial communities, potentially usable to counteract *Campylobacter* colonization of broiler caeca for the benefit of food safety. 

## 5. Conclusions

Novel approaches aimed at restoring broiler’s microbial barrier during *Campylobacter* infection might result from the findings of the present study. This might help develop new control strategies in order to mitigate the risk of *C. jejuni* colonization of broiler chickens and thus result in a lower food safety risk to human consumers of chicken meat.

## Figures and Tables

**Table 1:** *Campylobacter* infection dynamics over time for the four sampled farms. The number of positive animals over the total sampled is reported for each time point (from days 7 to 35 after hatching). Green cells indicate *Campylobacter* negative sampling points while Orange cells indicate *Campylobacter* positive ones.

Farm	Day 7	Day 14	Day 21	Day 28	Day 35
**1**	0/16	0/16	0/16	0/16	0/16
**2**	0/16	0/16	0/16	0/16	0/16
**3**	0/16	0/16	0/16	7/16	16/16
**4**	0/16	5/16	16/16	16/16	16/16

## Data Availability

The data presented in this study are available on request from the corresponding author.

## Supplementary Materials

The following are available online at https://www.mdpi.com/2076-2607/9/2/221/s1, Table S1: Genus level OTUs abundances, Table S2: Order level OTUs abundances.
